# Single-tablet regimen is associated with reduced efavirenz withdrawal in antiretroviral naïve or switching for simplification HIV-infected patients

**DOI:** 10.7448/IAS.15.6.18094

**Published:** 2012-11-11

**Authors:** M Fabbiani, P Grima, M Prosperi, I Fanti, M Colafigli, M Zaccarelli, A D'Avino, A Mondì, A Borghetti, M Fantoni, R Cauda, S Di Giambenedetto

**Affiliations:** 1 Catholic University of Sacred Heart, Institute of Clinical Infectious Diseases, Rome, Italy; 2 S. Caterina Novella Hospital, Infectious Diseases Unit, Galatina, Italy; 3 College of Medicine, University of Florida, Emerging Pathogens Institute, Gainesville, USA; 4 National Institute for Infectious Diseases “Lazzaro Spallanzani”, Viral Immunodeficiency Unit, Rome, Italy

## Abstract

**Purpose of the study:**

Efavirenz (EFV) is still discussed for its high rate of interruption due to adverse event, in particular central nervous system side effects (CNS-SE). Aim of the study was to define if better drug formulations up to single tablet regimen (STR), including (EFV) plus NRTI backbone (tenofovir-emtricitabine), reduced the risk of interruption.

**Methods:**

From the database of two reference centers, patients starting any cART regimen including EFV+2 NRTI or switching to EFV+2 NRTI for simplification after virological suppression were selected. Probability of interruption by virological failure, side effects, CNS-SE and any cause were assessed with survival analysis and Cox proportional hazard model.

**Summary of results:**

Overall, 533 patients, starting EFV-containing regimen from May 1998 to March 2012, were included (51.2% naïve, 48.8% switched). Patients characteristics: males 70.7%, median age 39 years, injecting drug use (IDU) 11.2%, median nadir CD4 194/mmc, median CD4 at EFV start 305/mmc: 38.7% started BID regimen, 43.9% OD regimen and 17.4% STR. At survival analysis, the overall proportion of EFV interruption was 19.1% at 1 year and 33.0% at 3 years; interruption for virological failure were 2.8% and 7.4% and for toxicity 10.2% and 15.9%, respectively. CNS-SE accounted for about half of interruptions for toxicity (5.7% and 8.0% at 1 and 3 years, respectively). Naïve patients had a higher risk of interruption as compared to switched patients: 37.7% vs. 28.0% at 3 years (p=0.06). While no significant difference was observed comparing OD vs. B ID regimens, starting with STR was associated with significant lower proportion of overall interruption at 3 years (17.1% vs. 36.6%, p<0.01). No virological failure was observed with STR up to 3 years (0.0% vs. 8.9%, p=0.05); no difference of interruption by overall toxicity and higher, though non-significant, frequency of interruption by CNS-SE (12.8% vs. 6.8%) were also observed. STR also accounted for lower proportion of interruption by patient wish, including low adherence (1.5% vs. 12.3%, p=0.01). At adjusted Cox model, STR (HR: 0.44; 95% CI: 0.26–0.77) and male gender (HR: 0.71; 95% CI: 0.53–0.97) were associated with lower risk of EFV interruption and IDU with higher risk (HR: 1.64; 95% CI: 1.11–2.42).

**Conclusions:**

In our experience, EFV co-formulated in STR was associated with lower virological failure and higher adherence, despite keeping CNS toxicity, thus reducing the risk of treatment interruption.

